# Different Kinetics of Perioperative CRP after Hip Arthroplasty for Elderly Femoral Neck Fracture with Elevated Preoperative CRP

**DOI:** 10.1155/2018/2140105

**Published:** 2018-04-24

**Authors:** Seung-Jae Lim, Kyung-Hwa Choi, Jin Hyuck Lee, Joon Young Jung, Woosol Han, Byung Hoon Lee

**Affiliations:** ^1^Department of Orthopaedic Surgery, Samsung Medical Center, Sungkyunkwan University School of Medicine, Seoul, Republic of Korea; ^2^Department of Preventive Medicine, Dankook University College of Medicine, Cheonan, Republic of Korea; ^3^Department of Emergency Medicine, School of Medicine, Kangwon National University, Kangwon, Chuncheun 200-701, Republic of Korea; ^4^Department of Orthopaedic Surgery, Kang-Dong Sacred Heart Hospital, Hallym University Medical Center, Seoul, Republic of Korea

## Abstract

This study aimed to determine the kinetics of four inflammatory markers and to identify the variables that affect the natural kinetics of inflammatory markers in aged patients having hip fractures with and without elevated preoperative CRP. 240 elderly patients who have been operated on for femoral neck fracture with no infectious complications were divided into two groups on elevated preoperative CRP level (>10 mg/L). The temporal values of four inflammatory markers of WBC, neutrophil count (*N*) (%), ESR, and CRP were assessed eight times every other day until the 14th postoperative day. At 48–60 h postoperatively, mean CRP was markedly higher in patients with preoperatively elevated CRP than in those with nonelevated CRP (122.1 ± 65.9 and 73.7 ± 35.5, *p* < 0.001). However, the abrupt elevation of CRP in the elevated group was conversely decreased on the 4th-5th postoperative day, demonstrating similar kinetic curves with no significant differences between both groups. For WBC, *N* (%), and ESR, both groups showed similar patterns of temporal values 14 days after surgery regardless of preoperative CRP level. Our findings could be used as guidelines for patient discharge and during the follow-up period after surgery.

## 1. Introduction

Arthroplasty is strongly recommended for elderly patients with unstable (displaced) femoral neck fracture [1]. In a subset of patients, delayed treatment due to several reasons can lead to higher mortality from concomitant medical problems. Several studies have indicated the advantage of surgery within 48 h, supporting that hip fracture surgery within 48 h of admission is associated with better outcomes [1]. However, an elevated level of C-reactive protein (CRP) can be a basis for delaying surgery, and it is one of the criteria for periprosthetic joint infection (PJI) diagnosis after surgery [2].

In elderly patients, femoral neck fractures usually occur following a fall, which can affect general health conditions with pulmonary or urinary infections, resulting in elevated preoperative CRP. In addition, a minimal elevation of CRP in a healthy, aged person might occur secondary to an increase in interleukin-6 gene expression that is related to frailty [3, 4]. Moreover, the level of preoperative CRP can be elevated from fracture itself or elevated in various clinical situations, such as concomitant cardiac disease, without an obvious infection [5].

If the elevated CRP level is caused by factors other than infection, arthroplasty does not need to be delayed simply because of the elevated preoperative CRP level [6]. On the other hand, if the reference level of preoperative CRP is elevated, this can affect the changes in temporal values of inflammatory markers including CRP and erythrocyte sedimentation rate (ESR) [7]. This may cause confusion during the follow-up period after surgery when determining the need for additional diagnostic procedures to confirm the presence of PJI.

In the postoperative stage, the use of antibiotics decreases the infection rate in total joint arthroplasty [8]; however, it is recommended that the duration of antibiotic prophylaxis should not exceed 24 h postoperatively because of an increased risk of resistance and toxicity [9]. In this sense, understanding the natural kinetics of inflammatory markers after surgery would be helpful for surgeons to determine the duration of antibiotic treatment and can assist in the early detection and monitoring of PJI.

The purpose of this study was to determine whether the perioperative kinetics of four inflammatory markers, white blood cell count (WBC), neutrophil count (*N*) (%), ESR, and CRP, have different patterns in aged patients having hip fractures with and without elevated preoperative CRP, additionally to identify the variables that affect the natural kinetics of inflammatory markers. We hypothesized that preoperative CRP level would affect the changes in temporal values of inflammatory markers after surgery and there would be patients' variables affecting the kinetics.

## 2. Materials and Methods

This retrospective study included 259 consecutive elderly patients who have been operated on for femoral neck fracture. They were divided into groups of elevated preoperative CRP level (>10 mg/L) and without elevated preoperative CRP level. The medical records of 289 hip hemiarthroplasties (HA) that were performed at our institution from January 2010 to June 2015 were reviewed. The inclusion criteria were as follows: a diagnosis of femoral neck fracture in patients aged above 60, patients admitted until stitches were removed on the 14th postoperative day, and patients followed up for more than 1 year. We excluded patients who had infectious complications of pneumonia (4 patients), PJI or wound infection (5 patients), urinary tract infection (6 patients), and death (4 patients). After these exclusions, 240 patients were identified as eligible for inclusion in this study.

Total patients were divided into two groups, 116 patients with elevated preoperative CRP (elevated CRP group) and 124 patients without elevated preoperative CRP (nonelevated CRP group) (Table 1). All patients or their proxy gave their informed consent to participate in the study, and this study was approved by the Institutional Review Board of our hospital.

A senior surgeon of our group performed all of the surgeries using the posterolateral approach [10]. Patients treated with other approaches or implants and those with pathological fractures were excluded. HA operation was performed in the lateral decubitus position using the posterolateral approach; then capsular repair and repair of the short external rotators were performed with strong nonabsorbable transosseous sutures in the greater trochanter. A Corail™ femoral stem (DePuy J&J, Warsaw, IN) with bipolar head was used in all cases. The incision was closed over a deep suction drain that was removed 48 h later. All patients received the same perioperative management with regard to anesthesia, multimodal analgesics, and wound management. The first wound dressing was placed on the second postoperative day, and wound dressings were routinely changed every other day. All patients were kept on physical (ankle pumps) and chemical prophylaxis for deep vein thrombosis (DVT) during their hospital stay.

The recorded data were reviewed with a focus on demographic characteristics, preexisting comorbidities, type of anesthesia used (general or regional), the duration of Foley insertion, time interval from injury to operation, cemented versus uncemented prosthesis, the number of blood transfusions, and operation time. For a CRP level that was higher than 10 mg/L, HA was performed if there were no clinical signs and symptoms of infectious processes during physical examinations. The clinical signs and symptoms of infectious conditions included fever (>37.3°C), redness, and local heat sensation. Preoperative infection diagnosis and treatment increased the delay before bipolar hemiarthroplasty from 0.7 to 8.3 days. Patients with elevated preoperative CRP were treated with the same protocol as others without elevated preoperative CRP. All patients received the same prophylactic antibiotics (2 g of cefazolin) within 1 h before incision. Intravenous antibiotics were also administered to all patients for 2 days. Allogenic blood transfusion was performed if hemoglobin level fell below 8.0 mg/dL or if anemic symptoms such as dyspnea or tachycardia persisted even after volume replacement in patients with a hemoglobin level between 7.0 and 8.0 mg/dL [11–13]. When transfusion was indicated, one unit of packed red blood cells was transfused at a time to increase the hemoglobin level to 9.0 g/dL. All participants received the recommended thromboprophylaxis regimen (with a low-molecular-weight heparin) from the operative day during 14 days after the operation. Tolerable weight-bearing ambulation was allowed on the second postoperative day after drainage removal.

We routinely evaluated preoperative WBC, *N* (%), CRP, and ESR on the day before surgery. Venous blood samples of all patients were obtained and checked eight times every other day until removal of staples on the 14th postoperative day. During the follow-up period, PJI was diagnosed using the criteria that were previously reported [14] and excluded from the enrolled database.

The current study obtained Institutional Review Board approval from our institution (KANGDONG 2016-11-006) before study onset, and our protocol was also approved. Informed consent was obtained from all participants.

### 2.1. Statistical Analysis

The temporal values of the four inflammatory markers were compared between the two groups. The values were represented as the mean and standard deviation at each time point. In addition, the changes in patterns of the markers were compared between the elevated and nonelevated CRP groups. The statistical significance of the differences between the two groups at each time point was determined using *t*-test. An a priori power analysis was performed to determine the sample size using the two-sided hypothesis test at an alpha level of 0.05, a power of 0.8, and repeated measures of ANOVA. Repeated measures of ANOVA were used to determine the within-subject effects of time (10 measurements) in two groups (i.e., patients with preoperatively elevated CRP values versus patients with nonelevated CRP values) for ESR and CRP. Sample size calculation was performed using G^*∗*^Power version 3.1.9.2 [15]. Continuous variables such as WBC, *N* (%), ESR, and CRP between the two groups (elevated CRP versus nonelevated CRP) were compared using two-tailed *t*-test. Body weight and height were measured, and BMI was calculated using the formula kg/m^2^ and categorized (<25, 25–29, and ≥30). Age was categorized as 60–69, 70–79, and ≥80. Categorical variables (i.e., age and BMI) between the two groups (elevated CRP versus nonelevated CRP) were compared using Pearson's Chi-square test. A generalized estimating equations model was used to analyze for repeated measures of the four markers through 14 days. The significance level was 0.05 in this study. All analyses were performed using R version 3.1.2 [16]. Regression analysis was performed to identify whether there are any significant factors that influence the temporal values of the four markers. Demographic variables included age, sex, BMI, preexisting comorbidities, anesthetic type, cemented versus uncemented prosthesis, and time interval from injury to operation.

## 3. Results

Demographic characteristics, including preexisting comorbidities, anesthesia type, cemented versus uncemented prosthesis, and time interval from injury to operation, are summarized in Table 1. The mean values with standard deviation of the four inflammatory markers, WBC, *N* (%), ESR, and CRP, for each time period are shown in Table 3. The above parameters were compared in patients with and without preoperatively elevated CRP, resulting in different kinetic curves only for CRP though an entire sampling day (*p* < 0.001). However, for WBC, *N* (%), and ESR, GEE analysis with repeated measurements could not determine statistically significant differences through an entire sampling day because of the interaction in time.

**Figure 1:**
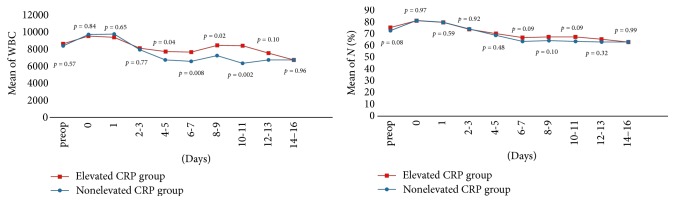
Perioperative WBC and neutrophil count (%) kinetics. *p* value estimated using *t*-test at each sampling day.

**Figure 2:**
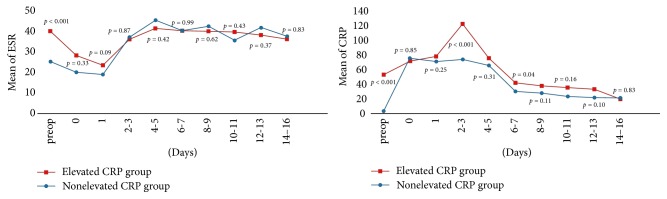
Perioperative ESR and CRP kinetics. *p* value estimated using *t*-test at each sampling day.

For WBC, *N* (%), and ESR, the two groups showed similar patterns of temporal values 14 days after surgery with no statistically significant differences regardless of preoperative CRP level. Preoperative WBC and *N* (%) showed no significant differences between the two groups (*p* = 0.57 and *p* = 0.08, resp.), and their kinetic curve patterns were relatively constant with time change (Figures 1 and 2). Preoperative ESR in patients with preoperatively elevated CRP was significantly higher than in those with nonelevated CRP (40.1 ± 25.7 and 25.4 ± 18.4, resp., *p* < 0.001). However, a similar pattern of ESR kinetics, elevated on the 3rd-4th postoperative day with a sustained curve, was observed 14 days after surgery in both groups (Figure 2).

At 48–60 h postoperatively, the mean CRP was markedly higher in patients with preoperatively elevated CRP that in those with nonelevated CRP (122.1 ± 65.9 and 73.7 ± 35.5, resp., *p* < 0.001). CRP kinetics after hip fracture surgery was different in patients with preoperatively elevated CRP compared with in those with nonelevated CRP. The abrupt elevation of CRP in patients with preoperatively elevated CRP was conversely decreased on the 4th-5th postoperative day, demonstrating similar kinetic curves with no significant differences between both groups (Figure 2). The difference in the kinetic curve for perioperative CRP between the two groups was influenced by male gender (*β* = −17.28, *p* < 0.001), age (*β* = 10.05, 7.28, *p* < 0.001), preexisting comorbidities ≥ 3 (*β* = −17.28, *p* < 0.001), and general anesthesia (*β* = 7.2, *p* < 0.001); on the other hand, it was not influenced by duration of Foley insertion (*p* = 0.18), cemented versus uncemented prosthesis (*p* = 0.39), time interval from injury to operation (*p* = 0.12), transfusion number (*p* = 0.10), and operation time (*p* = 0.87) (an appendix is available as Supplementary Materials Tables s1–4). The proportion of cases with CRP within normal ranges (<10 mg/L) at 14 days after surgery was lower in the elevated CRP group than in the nonelevated CRP group (38.7% and 59.6%, resp., *p* < 0.001) (Table 2).

## 4. Discussion

The principal finding of this study was (1) CRP kinetics after hip fracture surgery was different in patients with preoperatively elevated CRP compared with in those with nonelevated CRP (2) There was the abrupt elevation of CRP at 48–60 h postoperatively in patients with preoperatively elevated CRP was conversely decreased on the 4th-5th postoperative day, demonstrating similar kinetic curves with no significant differences between the elevated CRP and the nonelevated CRP groups.

The level of preoperative CRP and the temporal values of CRP and ESR after surgery have been used as guides to determine surgical timing and presence of PJI after surgery [17]. In this study, time interval from injury to operation was significantly longer in elevated CRP group. The wait time before surgery could be increased because of the evaluation of CRP progression or underlying pulmonary or urinary tract infection. However, a delay from injury to operation did not affect the CRP kinetics after operation. This should be noticed and also the delay should be minimized.

CRP level is an important inflammatory marker for developing treatment plans during the follow-up period after arthroplasty [18]. The preoperative level of CRP provides an individual reference level to compare the changes in CRP levels after surgery. However, CRP level can be elevated by intracapsular fractures of the femoral neck from increase in inflammatory factors because of synovial membrane production [19] and correlated with the severity of surgical or traumatic injury [20], underlying patients' conditions such as frailty [3, 4] or rheumatoid arthritis [7].

The reference levels of CRP and ESR have been criticized because of their low specificity for diagnosing infections [21]. Therefore, most surgeons refer to temporal patterns rather than the value at a specific time point [18]. Several studies have investigated CRP kinetics following surgical procedures [22–26]. These studies consistently found that CRP increases after the surgery, peaks on the second day, and then gradually returns to normal around the seventh day. When considering CRP kinetics, White et al. [24, 27] suggested that if the natural CRP kinetics is interrupted by a second rise or is persistently elevated, an infection should be suspected.

An understanding of postoperative CRP kinetics would contribute to the screening and diagnosis of PJI. However, no studies have investigated the changes in temporal values of inflammatory markers in relation to the reference level of preoperative CRP in patients with no infectious complications of pneumonia, urinary tract infection, and PJI. It is essential to rule out postoperative infection for shorter hospital stays after an operation. Therefore, it would be helpful if there are parameters that can function as guidelines to determine infection when deciding patient discharge, as the duration of antibiotic prophylaxis is restricted to not exceed 24 h postoperatively [9]. In this regard, a sensitive marker of CRP that is rapidly detectable might be a valuable and convenient parameter.

A higher peak value of mean CRP was observed 48–60 h postoperatively in patients with elevated preoperative CRP than in the other group, which gradually decreased to baseline levels and patterns within normal ranges around 3-4 days after surgery. In the present study, the proportion of cases with CRP within normal ranges (<10 mg/L) 14 days after surgery was still substantially lower in the elevated CRP group than in the nonelevated CRP group. This finding should be taken into consideration during the follow-up period.

If a sudden rise of CRP in the blood test of patients is confirmed 2-3 days after surgery, surgeons can encounter difficulties. The results of this study might provide a reference for the ongoing blood test with confidence. Our findings would also serve as a reference in determining the duration of prophylactic antibiotic treatment even for patients with preoperatively elevated CRP; a higher preoperative CRP level and a high peak value of CRP postoperatively might be an issue when surgeons decide to discontinue antibiotics treatment.

For WBC, *N* (%), and ESR, the two groups had similar patterns of time values 14 days after surgery with no statistically significant differences regardless of preoperative CRP level. Preoperative WBC and *N* (%) were not different between the two groups, and kinetics curves demonstrated relatively constant patterns with a high baseline, probably secondary to an increase in interleukin-6 gene expression and postulated to be related to frailty and predisposition to certain diseases at an advanced age even without signs of illness or inflammation [28].

Lastly, we found several variables affecting the difference in the kinetic curve for perioperative CRP in aged patients having hip fractures with and without elevated preoperative CRP. Male gender, older age, preexisting comorbidities ≥ 3, and general anesthesia were significantly influenced to the difference. Though we could not clarify the reason and did not evaluate other clinical variables such as fracture type, soft tissue damage, and inflammatory disease, the results might be useful to be referred in interpretation of the changes in temporal values of CRP after surgery.

Our study had an inherent limitation because of its retrospective design. Our findings were not able to provide conclusive data on the kinetics of the four inflammatory markers with regard to PJI because this study involved patients with no perioperative infections. This result would be helpful as guidelines for deciding patient discharge and the duration of antibiotic prophylaxis. Another limitation was elevated CRP was set arbitrally to greater than 10 or not even. The normal values of CRP levels were set to less than 0.5 mg/dL [16, 29]. However, CRP level suggesting chronic PJI were generally considered to be more than 10 mg/L [30–32]. Also, the main result of this study was not the threshold of CRP level to determine PJI but natural kinetics of CRP level with no PJI.

The main strength of our study was the large number of patients and its clinical relevance for postoperative blood sampling timing following hip arthroplasty for femoral neck fracture in elderly patients. And the consideration for rather homogenous cohort with only patients with isolated femoral neck fracture underwent bipolar hemiarthroplasty might compensate the statistical weakness.

In conclusion, CRP levels could be used as guidelines for patient discharge and during the follow-up period after surgery but have to be monitored for at least postoperative 14 days with consideration of the differential kinetic curves of perioperative CRP with a different reference level of preoperative CRP.

## Figures and Tables

**Table 1:** Patient demographics and baseline characteristics.

	Elevated CRP group (CRP > 10 mg/L)	Nonelevated CRP group (CRP ≤ 10 mg/L)	*p* value
Number	116	124	
Age, *n* (%)			0.06
60–69	13 (11.2%)	28 (22.6%)	
70–79	52 (44.8%)	48 (38.7%)	
≥80	51 (44%)	48 (38.7%)	
Sex; Female, *n* (%)	86 (74.1%)	100 (80.6%)	0.29
BMI^*∗*^ (kg/m^2^)	22.2 ± 3.8	22.8 ± 3.5	0.23
Preexisting comorbidities			
Hypertension	80 (69%)	83 (66.9%)	0.84
Diabetes	64 (55.2%)	73 (58.9%)	0.65
Number of preexisting comorbidities ≥ 3	41 (35.3%)	50 (40.3%)	0.51
General anesthesia, *n* (%)	64 (55.2%)	61 (49.2%)	0.43
Cemented type implant *n* (%)	29 (25%)	28 (22.6%)	0.77
Duration of Foley insertion, *d*^*∗*^	4.6 ± 5.7	2.8 ± 4.9	**0.04**
Time interval from injury to operation, *d*^*∗*^	4.3 ± 1.4	3 ± 1.4	**0.008**
Blood transfusion (pint)	3.5 ± 2.4	3.2 ± 2.2	0.395
Operation time (min)	103.7 ± 31.8	108.1 ± 31.3	0.252
Preoperative lab^*∗*^			
WBC	8596.2 ± 2869.1	8374.6 ± 3218.6	0.57
*N*%	75.7 ± 8.9	73.3 ± 12.3	0.08
ESR	40.1 ± 25.7	25.4 ± 18.4	**<0.0001**
CRP	53 ± 38.2	3.5 ± 6.7	**<0.0001**

^*∗*^Values are expressed as mean ± standard deviation. BMI, body mass index; CRP, C-reactive protein; ESR, erythrocyte sedimentation rate; *N*%, neutrophil count; WBC, white blood cell count; *p* value estimated using *t*-test or chi-square test. Values of *p* < 0.05 are displayed in bold.

**Table 2:** The proportion of the hip with the CRP value within normal ranges (<10 mg/L) at 14 days.

At POD 14 days	Elevated CRP group	Nonelevated CRP group	*p* value
CRP > 10 at postop 14 d	43 (62.3%)	38 (40.4%)	<0.001
CRP ≤ 10 at postop 14 d	26 (38.7%)	56 (59.6%)	<0.001

POD, postoperative day; CRP, C-reactive protein; *p* value estimated using chi-square test.

**Table 3:** The temporal value of the perioperative four inflammatory markers of WBC, *N* (%), CRP, and ESR.

Sampling day	WBC			*N*%			ESR			CRP		
	Elevated CRP group	Nonelevated CRP group	*p* value	Elevated CRP group	Nonelevated CRP group	*p* value	Elevated CRP group	Nonelevated CRP group	*p* value	Elevated CRP group	Nonelevated CRP group	*p* value
	Mean (SD)	Mean (SD)		Mean (SD)	Mean (SD)		Mean (SD)	Mean (SD)		Mean (SD)	Mean (SD)	
Preop	8596.2 (2869.1)	8374.6 (3218.6)	0.57	75.7 (8.9)	73.3 (12.3)	0.08	40.1 (25.7)	25.4 (18.4)	<0.001	53 (38.2)	3.5 (6.7)	<0.001
0	9595.9 (3328.5)	9736.8 (6978.9)	0.84	81.6 (7.6)	81.6 (6.6)	0.97	28.2 (24.4)	20.2 (25.3)	0.33	72.1 (52.9)	75.8 (55.6)	0.85
1	9379.4 (2866)	9776.7 (9050)	0.65	80.2 (6.5)	80.6 (5.3)	0.59	23.4 (17.8)	19.1 (17)	0.09	77.7 (38.4)	71 (37.7)	0.25
2-3	8096.7 (2757)	7938.5 (2999)	0.77	74.4 (7.8)	74.5 (9)	0.92	36.1 (24.6)	37.1 (28.2)	0.87	122.1 (65.9)	73.7 (35.5)	<0.001
4-5	7736.8 (2746.9)	6724.9 (2794.2)	0.04	70.5 (9.4)	69.3 (9.7)	0.48	41.4 (23.6)	45.4 (25.9)	0.42	75.3 (51.8)	65.6 (45.1)	0.31
6-7	7661.5 (2783.2)	6618.5 (2196.5)	0.008	67.2 (12.7)	64.1 (10)	0.09	40.3 (26.5)	40.4 (22.3)	0.99	42 (32.6)	30.5 (34.1)	0.04
8-9	8438.3 (3055)	7236.4 (2518.4)	0.02	68 (10.4)	64.6 (11.8)	0.1	39.9 (24.2)	42.3 (24.3)	0.62	38 (37.8)	27.8 (24.8)	0.11
10-11	8399.5 (3692.2)	6378.4 (2058.4)	0.002	67.7 (10.8)	63.7 (11)	0.09	39.7 (24.4)	35.7 (20.2)	0.43	35.7 (40)	23.2 (38.5)	0.16
12-13	7536.3 (2670.5)	6755.5 (2293.8)	0.1	65.9 (9.3)	63.8 (13)	0.32	38.1 (20.2)	41.8 (21.6)	0.37	33.4 (42.1)	21.8 (25.1)	0.1
14–16	6774.5 (2150.6)	6747.7 (2513.3)	0.96	63.6 (11.5)	63.6 (12.6)	0.99	36.3 (27.4)	37.5 (20.8)	0.83	20.4 (23.7)	21.7 (32.5)	0.83

SD; standard deviation; *p* value estimated using *t*-test.

## Data Availability

The datasets used and/or analyzed during the current study available from the corresponding author on reasonable request.

## Abbreviations

CRP: C-reactive protein
ESR: Erythrocyte sedimentation rate
WBC: White blood cell count
*N*: Neutrophil count
HA: Hemiarthroplasty.

## Supplementary Materials

Supplementary Material Table s1: effect on preoperative WBC through 14–16 days after surgery according to elevated CRP group by generalized estimating equation (GEE) model. Table s2: effect on preop *N*% through 14–16 days after surgery according to elevated CRP group by generalized estimating equation (GEE) model. Table s3: effect on preop ESR through 14–16 days after surgery according to elevated CRP group by generalized estimating equation (GEE) model. Table s4: effect on preop CRP through 14–16 days after surgery according to elevated CRP group by generalized estimating equation (GEE) model.
