# Supplementary material for: Different Kinetics of Perioperative CRP after Hip Arthroplasty for Elderly Femoral Neck Fracture with Elevated Preoperative CRP
Source: Biomed Res Int. 2018 Apr 24;2018:2140105. doi: 10.1155/2018/2140105 (PMC5941767; doi:10.1155/2018/2140105)
Supplement: Supplementary Material — Table s1: effect on preoperative WBC through 14–16 days after surgery according to elevated CRP group by generalized estimating equation (GEE) model. Table s2: effect on preop N% through 14–16 days after surgery according to elevated CRP group by generalized estimating equation (GEE) model. Table s3: effect on preop ESR through 14–16 days after surgery according to elevated CRP group by generalized estimating equation (GEE) model. Table s4: effect on preop CRP through 14–16 days after surgery according to elevated CRP group by generalized estimating equation (GEE) model. [file 2140105.f1.docx]

Supplementary

Table s1. Effect on preoperative WBC through 14-16 days after surgery according to Elevated CRP group by generalized estimating equation (GEE) model

|  | β | SE | p-value |
| --- | --- | --- | --- |
| (Intercept) | 8688.44 | 428.77 | <0.001 |
| Sampling day (vs. preop) |  |  |  |
| 0 | 999.63 | 407.16 | 0.01 |
| 1 | 797.85 | 384.40 | 0.04 |
| 2-3 | -427.12 | 433.37 | 0.32 |
| 4-5 | -882.51 | 417.55 | 0.04 |
| 6-7 | -926.61 | 403.25 | 0.02 |
| 8-9 | -189.09 | 481.90 | 0.70 |
| 10-11 | -261.37 | 608.95 | 0.67 |
| 12-13 | -1075.81 | 442.51 | 0.02 |
| 14-16 | -1886.76 | 414.89 | **<0.001** |
| Elevated CRP group vs. nonelevated CRP group | -184.90 | 395.95 | 0.64 |
| Female vs. male | -372.47 | 195.73 | 0.06 |
| Age group (vs. 60-69 years) |  |  |  |
| 70-79 | 295.30 | 255.34 | 0.25 |
| ≥80 | 279.89 | 301.52 | 0.35 |
| Number of comorbidity ≥ 3 | -790.29 | 201.79 | <0.001 |
| General anesthesia | 154.95 | 222.67 | 0.49 |
| BMI (kg/m^2^), vs. <25 |  |  |  |
| 25-<30 | 387.93 | 263.12 | 0.14 |
| ≥30 | -589.10 | 441.98 | 0.18 |
| Foley removal days | -8.03 | 8.54 | 0.35 |
| Op interval | 21.10 | 21.70 | 0.33 |
| Cemented | 91.77 | 194.80 | 0.64 |
| Transfusion number | -2.18 | 0.05 | 0.56 |
| Operation time | 352.69 | 299.36 | 0.49 |
| opday*elevated group | 360.52 | 796.00 | 0.65 |
| day1*elevated group | 608.57 | 955.85 | 0.52 |
| day2-3*elevated group | -83.20 | 679.96 | 0.90 |
| day4-5*elevated group | -701.23 | 613.89 | 0.25 |
| day6-7*elevated group | -804.54 | 551.73 | 0.15 |
| day8-9*elevated group | -867.89 | 645.90 | 0.18 |
| day10-11*elevated group | -1724.32 | 742.07 | **0.02** |
| day12-13*elevated group | -440.98 | 605.14 | 0.47 |
| day14-16*elevated group | 275.98 | 607.44 | 0.65 |

β, SE(standard error), and p-value estimated using GEE model

Values of P < 0.05 are displayed in bold.

Table s2. Effect on N% preop through 14-16 days after surgery according to Elevated CRP group by generalized estimating equation (GEE) model

|  | β | SE | p-value |
| --- | --- | --- | --- |
| (Intercept) | 74.87 | 1.25 | <0.001 |
| Sampling day (vs. preop) |  |  |  |
| 0 | 5.89 | 1.08 | <0.001 |
| 1 | 4.41 | 1.03 | <0.001 |
| 2-3 | -1.28 | 1.26 | 0.31 |
| 4-5 | -5.74 | 1.38 | <0.001 |
| 6-7 | -8.45 | 1.59 | <0.001 |
| 8-9 | -7.67 | 1.58 | <0.001 |
| 10-11 | -8.01 | 1.78 | <0.001 |
| 12-13 | -9.43 | 1.51 | <0.001 |
| 14-16 | -11.98 | 1.96 | <0.001 |
| Elevated CRP group vs. nonelevated CRP group | -2.08 | 1.36 | 0.13 |
| Female vs. male | -1.68 | 0.58 | 0.004 |
| Age group (vs. 60-69 years) |  |  |  |
| 70-79 | 1.55 | 0.76 | **0.04** |
| ≥80 | 3.92 | 0.79 | **<0.001** |
| Number of comorbidity ≥ 3 | 0.37 | 0.51 | 0.46 |
| General anesthesia | 0.46 | 0.50 | 0.35 |
| BMI (kg/m^2^), vs. <25 |  |  |  |
| 25-<30 | -0.92 | 0.54 | 0.09 |
| ≥30 | 0.38 | 1.11 | 0.73 |
| Foley removal days | 0.02 | 0.02 | 0.40 |
| Op interval | -0.21 | 0.08 | **0.01** |
| Cemented | 1.49 | 0.57 | **0.01** |
| Transfusion number | 0.65 | 0.52 | 0.69 |
| Operation time | 0.25 | 0.42 | 0.49 |
| opday*elevated group | 2.45 | 1.64 | 0.13 |
| day1*elevated group | 2.98 | 1.58 | 0.06 |
| day2-3*elevated group | 2.33 | 2.04 | 0.25 |
| day4-5*elevated group | 1.88 | 2.09 | 0.37 |
| day6-7*elevated group | -0.54 | 2.20 | 0.81 |
| day8-9*elevated group | -1.06 | 2.39 | 0.66 |
| day10-11*elevated group | -1.36 | 2.59 | 0.60 |
| day12-13*elevated group | -0.10 | 2.43 | 0.97 |
| day14-16*elevated group | 2.32 | 2.80 | 0.41 |

β, SE(standard error), and p-value estimated using GEE model

Values of P < 0.05 are displayed in bold.

Table s3. Effect on ESR preop through 14-16 days after surgery according to Elevated CRP group by generalized estimating equation (GEE) model

|  | β | SE | p-value |
| --- | --- | --- | --- |
| (Intercept) | 43.33 | 3.39 | <0.001 |
| Sampling day (vs. preop) |  |  |  |
| 0 | -12.65 | 5.97 | 0.03 |
| 1 | -17.05 | 3.09 | <0.001 |
| 2-3 | -3.50 | 4.16 | 0.40 |
| 4-5 | 0.76 | 4.22 | 0.86 |
| 6-7 | -0.39 | 3.92 | 0.92 |
| 8-9 | -1.07 | 4.10 | 0.79 |
| 10-11 | -0.60 | 4.48 | 0.89 |
| 12-13 | -3.17 | 3.71 | 0.39 |
| 14-16 | -4.78 | 4.83 | 0.32 |
| Elevated CRP group vs. nonelevated CRP group | -14.02 | 3.07 | **<0.001** |
| Female vs. male | -4.86 | 1.76 | 0.01 |
| Age group (vs. 60-69 years) |  |  |  |
| 70-79 | 1.84 | 1.87 | 0.32 |
| ≥80 | -1.95 | 1.94 | 0.31 |
| Number of comorbidity ≥ 3 | -3.69 | 1.36 | **0.01** |
| General anesthesia | -2.29 | 1.33 | 0.09 |
| BMI (kg/m^2^), vs. <25 |  |  |  |
| 25-<30 | 5.77 | 1.57 | **<0.001** |
| ≥30 | -4.12 | 3.49 | 0.24 |
| Foley removal days | -0.05 | 0.06 | 0.47 |
| Op interval | 0.53 | 0.16 | **0.001** |
| Cemented | -1.37 | 1.77 | 0.44 |
| Transfusion number | 2.12 | 0.05 | 0.21 |
| Operation time | 0.67 | 0.65 | 0.31 |
| opday*elevated group | 7.57 | 8.47 | 0.37 |
| day1*elevated group | 10.56 | 3.97 | **0.01** |
| day2-3*elevated group | 15.36 | 6.33 | **0.02** |
| day4-5*elevated group | 19.38 | 5.60 | **<0.001** |
| day6-7*elevated group | 14.18 | 5.00 | **0.00** |
| day8-9*elevated group | 17.93 | 5.43 | **<0.001** |
| day10-11*elevated group | 9.66 | 5.75 | 0.09 |
| day12-13*elevated group | 19.79 | 4.98 | **<0.001** |
| day14-16*elevated group | 16.57 | 5.93 | **0.01** |

β, SE(standard error), and p-value estimated using GEE model

Values of P < 0.05 are displayed in bold.

Table s4. Effect on CRP preop through 14-16 days after surgery according to Elevated CRP group by generalized estimating equation (GEE) model

|  | β | SE | p-value |
| --- | --- | --- | --- |
| (Intercept) | 49.43 | 5.12 | <0.001 |
| Sampling day (vs. preop) |  |  |  |
| 0 | 16.82 | 11.91 | 0.16 |
| 1 | 24.26 | 5.13 | <0.001 |
| 2-3 | 70.08 | 9.17 | <0.001 |
| 4-5 | 20.67 | 7.61 | 0.01 |
| 6-7 | -10.59 | 5.04 | 0.04 |
| 8-9 | -14.67 | 6.00 | 0.01 |
| 10-11 | -16.41 | 7.10 | 0.02 |
| 12-13 | -18.18 | 6.99 | 0.01 |
| 14-16 | -32.08 | 5.44 | <0.001 |
| Elevated CRP group vs. nonelevated CRP group | -45.07 | 3.47 | <0.001 |
| Female vs. male | -17.28 | 3.08 | **<0.001** |
| Age group (vs. 60-69 years) |  |  |  |
| 70-79 | 10.05 | 2.95 | **<0.001** |
| ≥80 | 10.62 | 3.08 | **<0.001** |
| Number of comorbidity ≥ 3 | -5.32 | 2.20 | **0.02** |
| General anesthesia | 7.20 | 2.20 | **0.00** |
| BMI (kg/m^2^), vs. <25 |  |  |  |
| 25-<30 | 0.17 | 2.53 | 0.95 |
| ≥30 | 4.42 | 8.48 | 0.60 |
| Foley removal days | 0.16 | 0.12 | 0.18 |
| Op interval | 0.54 | 0.34 | 0.12 |
| Cemented | 2.28 | 2.63 | 0.39 |
| Transfusion number | 3.31 | 2.05 | 0.10 |
| Operation time | 1.25 | 1.24 | 0.87 |
| opday*elevated group | 51.73 | 17.91 | **0.004** |
| day1*elevated group | 44.01 | 6.50 | **<0.001** |
| day2-3*elevated group | 1.02 | 11.01 | **<0.001** |
| day4-5*elevated group | 42.44 | 9.65 | **<0.001** |
| day6-7*elevated group | 36.63 | 6.47 | **<0.001** |
| day8-9*elevated group | 39.55 | 6.87 | **<0.001** |
| day10-11*elevated group | 36.28 | 9.36 | **<0.001** |
| day12-13*elevated group | 37.10 | 7.81 | **<0.001** |
| day14-16*elevated group | 50.19 | 6.99 | **<0.001** |

β, SE(standard error), and p-value estimated using GEE model

Values of P < 0.05 are displayed in bold.
